# Understanding Healing From Psychological Birth Trauma: A Lived Experience Perspective

**DOI:** 10.1111/jmwh.70052

**Published:** 2025-11-17

**Authors:** Lisa Middleton

**Affiliations:** ^1^ School of Social Work, Carleton University Ottawa Ontario Canada; ^2^ Midwifery Education Program Toronto Metropolitan University Toronto Ontario Canada

**Keywords:** healing, psychological birth trauma, postpartum, posttraumatic stress, qualitative research, recover

## Abstract

**Introduction:**

Psychological birth trauma affects a significant proportion of birthing individuals globally, with estimates ranging from 18% to 45% perceiving their birth as traumatic and 4% being diagnosed with posttraumatic stress disorder. Despite growing recognition of birth trauma, the lived experience of healing from it remains understudied.

**Methods:**

This qualitative study employed interpretive phenomenological analysis (IPA) to explore how 11 participants, purposively sampled for diverse birth trauma experiences, understood healing from birth trauma. Semistructured interviews were conducted, transcribed, and analyzed using IPA methodology.

**Results:**

Three main themes emerged: (1) healing as a process, not a destination; (2) healing as being at peace with the experience; and (3) healing as holding multiple truths. Participants described healing as an active, nonlinear process involving milestones, integration of the experience into daily life without being overwhelmed, and acceptance of changed priorities and emotions.

**Discussion:**

The findings highlight the importance of understanding trauma recovery as a gradual process, creating safer spaces for storytelling while respecting boundaries, and acknowledging the capacity to hold both challenging and positive emotions. The study calls for more research on birth trauma recovery centering the individual as an expert in their experience, involving diverse birthing individuals and researchers. Integrating lived experiences of healing is crucial for developing client‐centered initiatives and programming to support those affected by birth trauma.

## INTRODUCTION

Globally, there is growing recognition of psychological birth trauma and its impact on birthing individuals and their infants. It has been estimated 18% to 45% of people perceive their birth to be traumatic, and 4% of individuals are diagnosed with posttraumatic stress disorder (PTSD).^1^ Although there is no consensus on the definition of traumatic birth, Leinweber et al define it as “a woman's experience of interactions or events directly related to childbirth causing overwhelming distressing emotions and reactions; leading to short or long‐term negative impacts on a woman's health and well‐being.”^2^ ^(p691)^

**QUICK POINTS**

✦ Healing from birth trauma is an individualized, nonlinear process unfolding over time and through meaningful personal milestones.
✦ Participants emphasized the importance of making peace with their experiences rather than achieving a fixed state of recovery.
✦ The ability to hold multiple, sometimes conflicting, emotions was central to their understanding of healing.
✦ Midwives and support providers should foster safe, nonjudgmental spaces for storytelling and emotional processing.
✦ Centering the voices and expertise of birthing individuals is essential for developing equitable, trauma‐informed care and support systems.

Despite a growing understanding of birth trauma, research on healing from birth trauma remains limited. The emphasis on individual approaches to traumatic birth recovery^3^, ^4^, ^5^, ^6^ may not incorporate an understanding of trauma and trauma healing going beyond the *Diagnostic and Statistical Manual of Mental Disorder, Fifth edition* (DSM‐V) definition of trauma and trauma recovery. Recovery from PTSD is often understood as a reduction in posttraumatic symptoms, such as flashbacks, avoidance, and intrusive thoughts.^7^ This is often measured using pretreatment and posttreatment surveys such as the City Birth Trauma Scale,^4^ which is validated by the DSM‐V. However, there is still limited research examining how individuals perceive healing from birth trauma through the lens of their lived experiences.

A 2024 scoping review^8^ revealed that the limited number of qualitative studies on PTSD recovery primarily focused on military populations. A study by St Cyr et al^9^ found participants’ subjective experiences of recovery often did not align with their scores on standardized PTSD measures. In many cases, participants described feeling more recovered than their PTSD scale scores suggested, highlighting a disconnect between clinical assessments and lived experiences of healing. This demonstrates the importance of incorporating lived experience into the understanding of what healing and recovery from PTSD can look like. Although only a minority of individuals who experience birth trauma receive a formal PTSD diagnosis, many still exhibit posttraumatic stress symptoms.^4^, ^10^ As such, exploring how people make sense of PTSD recovery is relevant to understanding the needs and experiences of those affected by birth trauma.

Previous phenomenological research on birth trauma has focused on experiences of birth trauma among individuals^11^, ^12^, ^13^, ^14^, ^15^ and couples.^16^, ^17^ Although some studies have examined the experience of birth trauma and its impact on the postpartum period,^18^ phenomenological research has not yet been conducted on the experience of healing from birth trauma. Drawing from lived experience, this article sets out to demonstrate what birth trauma recovery means for individuals experiencing birth trauma. This article is part of a larger independent doctor of philosophy (PhD) research project exploring the experience of birth trauma and the experience of healing from birth trauma using interpretive phenomenological analysis (IPA) grounded in intersectional feminist trauma theory.

## METHODS

The current qualitative study used IPA to understand how participants experienced and understood birth trauma recovery. IPA methodology focuses on how an individual makes sense of a given experience.^19^, ^20^, ^21^, ^22^ IPA focuses on ideographic analysis (case by case) and does not strive to universalize the experience of a phenomenon.^20^ Furthermore, IPA pays particular attention to the researcher and how the researcher's social and historical life world impacts the analysis. It employs what Smith et al^20^ called *double hermeneutics*. This means the participant made sense of a particular experience, whereas the researcher made sense of the participant's experience.

### Participants

The study recruited 11 participants. To enhance diversity beyond the dominant demographic represented in birth trauma literature (ie, White, able‐bodied, cisgender, coparent women partnered with men), the study aimed to recruit at least 50% of participants who identified as LGBTQIA2+ (lesbian, gay, bisexual, trans, queer, intersex, asexual, 2‐spirit, or other), Black, Indigenous, racialized, disabled/person with a disability, trans/nonbinary, or single parents by choice. Nine of the 11 participants identified with at least one of these identities. Participants were selected to reflect the diversity of the Outaouais region (Canada).

Smith et al express that 10 participants is largely seen as the gold standard in IPA research. To participate, participants had to self‐identify as having experienced a psychologically traumatic birth and as having healed from a psychologically traumatic birth. Participants were recruited using purposive sampling in the Outaouais region in May 2024.

### Data Collection

The participants were invited to participate in 3 semistructured interviews (in‐person interview, follow‐up phone interview, and an optional focus group). Using an interview guide, interviews were approximately 90 minutes in length and audio recorded. The 2‐part interview included (1) making sense of the past: telling their birth story; and (2) making sense of the present: exploring healing from birth trauma.

### Data Analysis

Each interview was transcribed using Descript and subsequently verified by the researcher. A case‐by‐case approach was used, with each case study being fully analyzed before proceeding to the next. Transcripts were read and reread using a reflexive approach informed by intersectional feminist trauma theory, with particular attention to power dynamics and intersecting identities. Feminist phenomenology specifically encourages researchers to identify and highlight how dimensions such as race, gender, sexual orientation, and ability emerge in participants’ narratives.^23^ This was supported by a reflective journaling process in which I posed questions such as: *How is this participant's identity (if at all) present in their experience? How are power and privilege operating in this account?* These reflections were supported by direct quotations from the transcripts and validated through member checking.

Exploratory notes and experiential statements (themes) were made. Themes were arranged chronologically and checked using the participants’ words to ensure they were correct and reflected their experiences. Themes were then placed coherently on a table to compare the participants’ narratives. A draft case analysis was provided to the participants before the follow‐up phone interviews. Overwhelmingly, participants verified their case analyses.

Individual case analyses were then cross‐compared to identify the repeating themes. A draft data analysis was then discussed with the researcher's PhD research supervisor to ensure themes were appropriate and accurate. Prior to the focus group, draft data analysis was provided to the participants in the study. Notes from the final focus group were used to verify the data analysis and to add additional thoughts on what it meant to experience and heal from birth trauma. The final draft of the data analysis was sent to the researcher's thesis committee for review and discussion. Reported data adheres to the Standards for Reporting Qualitative Research (Supporting Information: Appendix S1).^24^

### Ethics

The Carleton University Research Ethics Board‐A approved this study. To maintain participant confidentiality, participants were given pseudonyms, and identifying information was removed. Written informed consent was obtained from participants at the outset of the research, with additional verbal consent secured prior to each interview. Written consent was also obtained before the optional focus group.

Although this study aimed to amplify and integrate a diverse range of birthing voices, I acknowledge my positionality shaped the research process. As a White, settler, Queer, feminist social worker and midwife and someone who has personally experienced psychological birth trauma, my analysis was inevitably influenced by these intersecting identities and experiences. I approached the data through this lens, engaging in reflexivity to remain critically aware of how my background could shape interpretation and meaning‐making. This included keeping a reflexive journal to critically examine my assumptions, monitor personal responses to participants’ narratives, and note any potential instances of transference throughout the research process. I regularly asked myself key questions like: *What is the participant's experience? What is my experience? What is the shared experience between us?* I also actively sought feedback from participants and colleagues and chose a thesis committee representive of diverse lived experiences such as race, culture, birth experiences, research disciplines, and research interests. Although this PhD project was an independent research project, in the future, having a diverse committee of researchers would be key in intersectional feminist trauma research.

## RESULTS

Healing from birth trauma is understood as the following: (1) healing is a process and not a destination, (2) healing is being at peace with your experience, and (3) healing is holding multiple truths. Each theme was integrated into the 11 participants’ experiences.

### Healing is a Process and Not a Destination

Healing from birth trauma is a process. Although some participants were explicit about recovery, most understood healing as a process occurring at various stages. Additionally, during the final focus group, participants expressed although participating in the study was at times challenging, telling their stories felt like part of their healing journey. Sarah discussed the process of healing from birth trauma:

I think there's multiple points, of healing, I remember being in the hospital bed after everything had happened, and thinking for the first time, you've done everything, you couldn't have done anything more. This was your all. That was the first step…I remember going to see my doctor after a month of work. My family doctor was like, “I'm so proud of you,” He said most people just probably, wouldn't do what you've done. Is it healing? I don't know, but it was first moment where I was like “It's gonna be okay”…It's been a long road, I think I've been healing the whole time, and I feel I've been making progress. [Sarah]

Healing is a long road with continued progress, meaning healing is an active process. Healing as a process also means learning and understanding the interconnection of the experience and how it has impacted you; Marie said,

I think healing means knowing you went through trauma and knowing this did impact you and how it impacted you. I think part of healing is understanding how those things are interconnected. Healing is identifying all those tentacles and being like, “oh, that's why I was like this, or yeah, that might have been made worse because of this.” I think I'm probably still healing because I'm still seeing the impacts of healing. [Marie]

Healing means uncovering and understanding new aspects of the self. In contrast, healed is static, meaning you have reached the point at which you are not able to heal anymore. Kate said,

There are milestones of healing. Healing? Yes. Healed? No. Not even physically…but when I embraced the idea of having another baby. When I was able to have another pregnancy and find joy in it, not fear. I think I was healed, or healed enough at that point, that I was able to joyfully move forward into new risk and new possibility. That would be a turning point. [Kate]

Healing refers to being able to move forward in life. Healing from birth trauma is not a linear process, and it will resurface at various moments and experiences. Lena said,

Healing, it's not linear and things are going to bring it back. Like my sister having to be induced for a premature rupture of membranes and I was like I hope this goes well. I hope she's well supported and not putting my own crap on it. [Lena]

Nonlinear means understanding you have the tools to be able to cope with your experience when it resurfaces. Healing also refers to moving to a place where it is not all‐consuming, all the time, and finding peace with the experience of birth trauma.

### Healing Is Being at Peace

Being at peace with birth trauma allows people to integrate their experiences into their lives. Although the trauma still impacts them, it does not carry the same weight, nor does it consume their daily thoughts and actions. Being at peace means letting go of the heaviness of experience. Amy said,

Healing feels, you're more at peace with it. I'm not feeling triggered or activated from either thinking about it or witnessing similar experiences…It doesn't feel as heavy and dark and it feels, like it's not with me. I've left it in the past and it happened, but I'm okay with it now…I can just kind of describe it, but it doesn't hold emotional heaviness that it did for me before. [Amy]

Being at peace means leaving it in the past. Being able to describe your story but not being consumed by it is an important distinction. This was also emphasized by Irene:

What would healing sound like? Probably a release of the weight. Of carrying all the things. Because I think there's a deep sadness. There's anger. There's shame. There's fear. There's a lot of things you carry when you're still in the trenches and I think recovery is being able to put all of that down and releasing it and knowing that it's okay for it to bubble up or be triggered or show up here and there, but to not have it be part of your daily life anymore and being able to be like “that was part of my story, but that's not my whole story.” And to be able to have a very valid reason for why you feel those things. To feel them, acknowledge them, and then release them. And know you can move past that part. [Irene]

Trauma is symbolized as heavy and all‐consuming, whereas healing is symbolized as putting heavy objects down and integrating the experience into life. Being at peace means talking about birth trauma and not being consumed; it means letting go of anger. Julie said,

I think it was when I could tell the story to somebody and feel like I could get through it where I had some emotional distance from it. I went through two periods: One where, I couldn't tell the story without sobbing and then, where I was like, “oh, everything was fine.” Niether of those things were particularly helpful or healthy. I think it was by the time I could say, “hey, this happened to me, it was bad” but I can talk about it and I'm at peace with what happened. [Julie]

Being at peace with what happened and being able to talk and discuss your experience. Identifying triggers and choosing not to engage are an important part of healing. For Lynn it meant,

Healing is being able to know those triggers within myself and know when I'm in a position that's not going to make me feel good and to not do that because I know that's going to make me feel unwell. That, to me, is healing. [Lynn]

Healing refers to identifying, integrating, releasing, and sitting in your experience, but not consuming. Healing also implies people hold multiple truths.

### Healing Is Making Space for Self and Holding Multiple Truths

Holding multiple truths involves recognizing and balancing the coexistence of what is acceptable and what is not. Healing involves holding multiple truths and feelings in mind. Sarah said,

I think it means being my own friend. Just being able to be kind to me. It means being able to make space for all the emotions and not being embarrassed or ashamed or guilty about them. Healing is learning how to be kind to myself and make space for the things I've gone through and learning to live with them in harmony and making space for them and not trying to get rid of them. [Sarah]

Healing is accepting what you are feeling and being okay with what you feel. This allows you to coexist with birth trauma. Nunu said, “I think just accepting the process and accepting the fact there were difficulties related to the birth. It does not mean because the birth was difficult, it was a failed process. I think that's the biggest shift.”

Healing is the understanding that, even when outcomes differ from you hoped, you have not failed. A challenging birth does not equate to failure. Holding multiple truths can involve acknowledging birth trauma may alter the future you envisioned for yourself. Maddy said,

I had imagined a much bigger family. I think being more realistic about our circumstances is a part of my healing. Like it's medically and physically, the right choice for me and my body, but even being able to entertain that conversation is healing because I was pretty stubborn about what I wanted my family to look like. So being able to adapt and understand and accept my circumstances is healing for me. [Maddy]

Grieving the loss of what was envisioned and holding that truth while being okay with moving on with a different plan is healing. For one participant in the sample, who was a labor and delivery nurse, holding multiple truths as a form of healing involved the decision to leave the nursing profession because of birth trauma. Lynn expressed this here:

I felt ready to get back to work too, because it had been 18 months and I loved my job so much. I was struggling with every delivery. I was going home crying; I was a disaster. I loved my job so, so much and that's why I've struggled, there's nowhere else I felt like working. I want to work there, and I want to get past that. I wanted to get past the trauma because I didn't want someone else to take away something that I enjoyed. Only in the last couple months have I given up on that. I'm just like, “fuck it. It's just not for me anymore. it's done.” I have nothing to prove. And I'm not going to continue doing it. It's done. [Lynn]

Trauma changes you, including wants, needs, and priorities, and being able to grieve and hold those truths is healing. Despite Lynn loving her job as a labor and delivery nurse, ultimately, she recognized that stepping away from her career was necessary for her mental and emotional well‐being. Healing also means holding multiple truths at once, including the capacity to carry complex emotions, needs, and desires. Salma said,

Recognizing I know that it's ok to have experienced the thing everyone else has experienced and they're all fine and you're not. And actually, that is ok. It's not a sign of weakness. Healing isn't being ok all the time. Healing is saying I'm not ok and I need help and to say it out loud. I am not ok…Healing is having a feeling of abundance within you. So, you can make room for everything, the happiness, the struggles and the boring and then fun, that's healing. [Salma]

Having the capacity to hold multiple truths is creating abundance within yourself and your life to fully and entirely accept yourself and move forward with self‐love.

## DISCUSSION

Although there is growing research on the experience of birth trauma and its causes, there has been a lack of focus on healing from birth trauma and what it means to heal from birth trauma. Formal research on recovery from birth trauma is often conducted in the biomedical field. The current study used qualitative IPA to explore the understanding of healing from lived experiences. For the participants in the study, healing from birth trauma is understood by 3 themes: (1) healing is a journey and not a destination, (2) healing is being at peace, and (3) healing is holding multiple truths.

Healing as a journey, not as a destination, understands that the process of healing is dynamic and not static. Participants could point to various milestones in their healing journey and often felt that despite feeling “healed,” they were in a constant state of healing. Although participants self‐identified as healed at the time of the study, this highlights the evolving nature of healing. It also opens opportunities for future research to explore how individuals’ understanding of healing changes over time.

Judith Herman^25^ discusses healing from trauma as a process. She says healing involves 3 parts: safety and stabilization, remembering and mourning, and reconnection and integration. This nonlinear process takes time for trauma survivors to move through. Understanding trauma recovery as a process rather than an end state has been expressed in trauma research focused on lived experiences^9^ as well as in research focused on posttraumatic growth.^26^ Knowing and acknowledging that healing from trauma is a gradual process may help normalize the nonlinear process.^8^

Being at peace involves the ability to integrate one's birth experience into daily life without being overwhelmed by associated trauma. Recovery from trauma emphasizes the development of self‐awareness and insight, which are often reported by individuals who experience posttraumatic growth.^27^ The integration of traumatic memories and the reduction of their emotional intensity is a central theme in the work of trauma theorists such as Lavine,^28^ Rotschild,^29^ Van der Kolk,^30^ and Herman.^25^ For individuals affected by birth trauma, understanding that healing may require setting boundaries between the telling and retelling of their birth stories is crucial. Recognizing elements that support or hinder mental health is a key component of self‐awareness. In addition, health care providers can play a supportive role by identifying posttraumatic stress in individuals who have given birth and by creating safer spaces for storytelling, while also promoting and respecting these boundaries.

Healing from birth trauma implies being able to hold space for multiple truths. For participants, this involved accepting and allowing priorities, wants, and needs to change because of the experience of birth trauma. Holding multiple truths also involves increasing the capacity to feel and experience a plethora of emotions. Research on posttraumatic growth following birth trauma highlights the capacity to simultaneously hold both challenging and positive emotions.^31^

The process of accepting and embracing new life trajectories as a result of the trauma was experienced as both a hindrance and a success in trauma recovery.^27^ This meant that although it was difficult for individuals to accept their lives had permanently changed due to birth trauma, like in Lynn and Maddy's accounts, coming to terms with their new life trajectories also represented their ability to move forward. Even though their new life paths differed significantly from their lives before the trauma, and they were not entirely happy with these changes, they have come to accept them as part of their healing journey.

### Strengths and Limitations

Although the study met the gold standard of IPA, small sample sizes are often understood as a limitation of IPA studies. Although the aim of IPA is not to generalize participants’ experiences, the limited sample may affect the transferability of the findings to broader research on birth trauma recovery. The existing literature on individuals’ lived experiences of trauma recovery reveals patterns similar to those observed in this study; the overall lack of research in this area can presents credibility challenges.

Data analysis followed the IPA methodology as outlined by Smith et al.^20^ Given that phenomenology involves interpreting individual experiences from the researcher's perspective, there is a possibility these interpretations may not fully align with the participants’ intended meanings. To enhance the credibility and accuracy of the findings, member checking was conducted individually and within the focus group setting.

This study focused on lived experiences to understand recovery from birth trauma. This approach can help expand our understanding of trauma and recovery from birth trauma. Although this can be a strength of the research, it can also be a limitation, as it does not use the DSM‐V definition of trauma and trauma recovery, nor did the study limit who could participate based on the DSM‐V criteria of trauma.

## CONCLUSION

Although the experience of birth trauma and the causation of birth trauma are becoming more widely understood, research on how people move forward from birth trauma is limited. This article specifically aimed to explore the meaning of healing from birth trauma through the lens of lived experience. The findings of this study draw attention to the various ways in which people understand healing from birth trauma. Integrating the lived experience of birth trauma recovery is important for creating and supporting client‐centered initiatives and programming. Furthermore, this article calls for more research on birth trauma recovery centering the individual as an expert in their experience and recovery. This includes purposively involving diverse birthing individuals and diverse researchers to highlight the experiences of those who have been systematically excluded from birth trauma research. This is important to ensure research on birth trauma and birth trauma recovery is person‐centered and inclusive of all individuals who have experienced psychological birth trauma.

## CONFLICT OF INTEREST

The author has no conflicts of interest to disclose.

## Supporting information




**Appendix S1**: Standards for Reporting Qualitative Research (SRQR)
